# Adolescents’ Aided Recall of Targeted and Non-Targeted Tobacco Communication Campaigns in the United States

**DOI:** 10.3390/ijerph15112363

**Published:** 2018-10-25

**Authors:** Sarah D. Kowitt, Allison J. Lazard, Tara L. Queen, Seth M. Noar, Adam O. Goldstein

**Affiliations:** 1 Department of Family Medicine, University of North Carolina at Chapel Hill, Chapel Hill, NC 27599, USA; adam_goldstein@med.unc.edu; 2 School of Media and Journalism, University of North Carolina at Chapel Hill, Chapel Hill, NC 27599, USA; lazard@unc.edu (A.J.L.); noar@unc.edu (S.M.N.); 3 Lineberger Comprehensive Cancer Center, University of North Carolina at Chapel Hill, Chapel Hill, NC 27599, USA; tlqueen@email.unc.edu

**Keywords:** tobacco prevention and control, communication campaigns, United States, adolescents

## Abstract

We examined whether advertisements from two national tobacco control campaigns targeting adolescents (i.e., *The Real Cost*, *Fresh Empire*) and one campaign targeting adults (i.e., *Tips from Former Smokers*) were reaching adolescents. Data came from a national sample of adolescents aged 13 to 17 years (*n* = 975) surveyed by phone from August 2016 to May 2017. We assessed recall and attitudes toward five specific advertisements and three campaign slogans and examined differences by sub-groups. Almost all (95%) adolescents recalled seeing at least one campaign advertisement. Aided recall of *The Real Cost* and *Tips from Former Smokers* slogans was high (65.5% and 71.6%, respectively), while aided recall of *Fresh Empire* slogan was lower (15.3%) (χ^2^ *p*-value: *p* < 0.001); however, Black adolescents had higher odds of recalling the *Fresh Empire* ad (aOR: 2.28, 95% CI: 1.39, 3.73) and slogan (aOR: 2.64, 95% CI: 1.06, 6.54) compared to White adolescents. Increased exposure to the advertisements (i.e., recalling more advertisements) was significantly associated with higher odds of reporting negative feelings toward tobacco products in 4/5 models (aORs from 1.34 to 1.61). Large-scale national campaigns can have wide reach among both targeted and non-targeted audiences with added benefits for cumulative cross-campaign exposure to advertisements.

## 1. Introduction

Adolescents are a priority target audience of tobacco education campaigns, as they are most at-risk for initiating tobacco use that could lead to a lifetime of nicotine addiction [1,2]. Despite lower rates of combustible cigarette use in recent years, every day thousands of adolescents smoke their first cigarette and many become daily tobacco users [3]—contributing to over 5 million adolescents at risk of premature death [1,2]. Increasing evidence of dual- and poly-use of tobacco products [4,5,6], coupled with problematic initiation rates among adolescents, presents an imminent need for continued tobacco control efforts, including national communication campaigns.

Previous researchers have extensively documented the effectiveness of tobacco communication campaigns on targeted audiences. For example, launched by the United States (US) Food and Drug Administration (FDA), *The Real Cost* campaign aims to educate at-risk teens about the harmful effects of tobacco use. The campaign was launched in 2014 through traditional broadcast (i.e., TV, radio), print, out-of-home (e.g., billboards), and digital advertising (e.g., website, social media) [7,8]. Specifically, the campaign was designed to prevent cigarette smoking initiation and decrease cigarette smoking experimentation by changing beliefs about: (1) the health consequences of tobacco use, (2) the loss of control and independence that are associated with tobacco use, and (3) the dangerous chemicals in cigarette smoke [8]. The ads therefore featured visceral portrayals of the health consequences of smoking and the loss of control and independence that result from smoking [8]. Research suggests that the campaign has reached almost 90% of adolescents [8], which is important since exposure and attention to the campaign are metrics [8,9,10,11,12] that serve as the critical first steps to impact attitude and behavior change [13,14,15]. Additionally, researchers have estimated that exposure to *The Real Cost* campaign was associated with preventing almost 350,000 adolescents aged 11–18 from initiating smoking during 2014–2016 [16].

Research has also established that adult-targeted tobacco communication campaigns can have impacts on non-targeted audiences (i.e., adolescents) and may have a larger impact for the reduction of tobacco-related harm than focusing only on preventing adolescents from tobacco use [17]. Developmentally, adolescents are often looking for ways to identify and be treated as adults. By making the adult activity unappealing, these campaigns may resonate with adolescents who are developing perceptions and behaviors that signify adulthood. Negative, visceral [18] anti-tobacco ads are also remembered by teens, and can have a positive impact on smoking-related beliefs and reductions in tobacco use [19]. Launched by the Centers for Disease Control and Prevention (CDC), the *Tips from Former Smokers* campaign profiles real smokers dealing with the life-long debilitating health consequences of tobacco use or exposure. *Tips from Former Smokers* launched in 2012 with TV, radio, print, and out-of-home (e.g., billboards), and digital ads [20]. The goals of *Tips from Former Smokers* were to: (1) build public awareness of the immediate health damage caused by smoking and exposure to secondhand smoke, (2) encourage smokers to quit and make free help available, (3) encourage smokers not to smoke around others, and (4) encourage nonsmokers to protect themselves and their families from exposure to secondhand smoke [20]. This campaign has had considerable reach and impact among adults, with researchers estimating that exposure to *Tips from Former Smokers* was associated with over 9 million additional quit attempts between 2012 and 2015 [21]. There is less evidence whether the campaign is salient among adolescents, although one study found high exposure among adolescents [22] and another found that the campaign was associated with adolescents’ increased intentions to quit smoking and lower susceptibility to smoking [23].

Not all campaigns are designed to have mass appeal, however, and some campaigns increase their effectiveness through targeting a very specific audience segment. By appealing to a segment of the population with commonly shared characteristics, such as lifestyle or culture, these campaigns can increase the likelihood that their intended audience will perceive the information as relevant and attend to or engage with the messages [12]. Campaigns designed to reach unique adolescent population segments may be more likely to resonate and have a lasting impact on health behavior [24,25]. At least two tobacco communication campaigns have been designed with these goals in mind. The *Fresh Empire* campaign (from the FDA) targets at-risk multicultural adolescents who identify with “hip-hop culture,” specifically African American, Hispanic, and Asian American/Pacific Islander adolescents [26]. The campaign was initially launch in May 2015 in four Southeast markets and expanded to reach 37 markets with TV, print, and digital advertising (e.g., website, social media) in October 2015 [27]. Specifically, this campaign encourages “hip-hop youth” to be successful, attractive, and in control by messaging about addiction, harmful chemicals in tobacco, and the health consequences of using tobacco. In addition, *The Real Cost* “Face of Dip” ad targets young, white, rural males at risk for using smokeless tobacco. While outcome evaluations of the *Fresh Empire* campaign and *The Real Cost* “Face of Dip” ad are currently underway, no results have been reported to date.

Determining whether these campaigns are having an impact on adolescents across the US—inclusive of the intended population segments (targeted audience) and audience spillover (non-targeted audience)—is a critical step to understand their potential contributions to tobacco control efforts. In light of previous research, this study examined aided recall of and responses to three ads from *The Real Cost* (two for which recall has not been reported in the literature previously: “Face of Dip” and “Science Class”). Further, we evaluated spillover effects of aided recall and responses to the *Tips from Former Smokers* campaign and to the more targeted *Fresh Empire* campaign and examined effects of cumulative exposure to campaigns.

## 2. Materials and Methods

### 2.1. Participants

The data collected were from a national sample of adolescents. From August 2016 to May 2017, the Carolina Survey Research Laboratory (CSRL) conducted phone surveys for a probability sample of 975 adolescents living in the US. Adult random digit dialing frames were used to recruit adolescents, ages 13–17, within the household. To supplement this sample, an electronic white page frame targeting households with adolescents was used. Interviewers conducted the survey in English or Spanish and adolescents were compensated $40 for their participation. The survey included questions on tobacco regulatory constructs (e.g., tobacco constituent perceptions, tobacco regulatory credibility). CSRL oversampled counties with higher prevalence of smokers and low-income respondents. Interviewers obtained verbal consent from adolescents’ parents or guardians and verbal assent from the adolescents. The response rate among adolescents was 32.8%, calculated using the American Association for Public Opinion Research Formula (4). The weighted sample is nationally-representative of 13–17 years old living in the US, with cell or landline access, who could expect to obtain consent from a guardian for a tobacco use phone survey. The Institutional Review Board at the University of North Carolina reviewed and approved study procedures (NO. 13-2779).

### 2.2. Measures

#### 2.2.1. Aided Recall of Campaign Slogans

We first assessed aided recall of tobacco communication campaign slogans by asking adolescents, “Have you ever seen or heard any ads on television or radio with the slogan…” [11]. Adolescents were then randomized to hear 1 of 3 slogans: *The Real Cost*, *Tips from Former Smokers*, or *Fresh Empire*. Responses included “yes” (coded as 1) and “no” (coded as 0). Participants were randomized to hear 1 of 3 slogans to reduce the response burden of the overall survey (instead of 1 person being asked about 3 slogans, 1 person would only be asked about 1 slogan).

#### 2.2.2. Aided Recall of Campaign Ads

We assessed aided recall of 5 campaign ads by asking: “In the last year, have you seen or heard any anti-smoking ads…” followed by:

1. …where a tiny man bullies a teenager? (“*The Real Cost*—Bully”) [11]
2. … where a scary, insect-like creature appears in a high school science class or under the bleachers? (“*The Real Cost*—Science Class”)
3. … about the harms of smokeless tobacco showing a young man with scars from mouth cancer? (“*The Real Cost*—Face of Dip”)
4. … where a former smoker talks about their serious health problems caused by smoking? (“*Tips from Former Smokers*—Serious Health Problems”)
5. … where a young person talks about rejecting smoking to keep it fresh for themselves and their family? (“*Fresh Empire*—Keeping It Fresh”)

For each of the five ads, adolescents were instructed to respond “yes” (coded as 1) or “no” (coded as 0). We created an ad recall index by summing the number of ads. Notably, since participants were randomized to one of the three campaign slogans for recall, participants only heard the campaign name that corresponded with one or three of the five described ads.

All ads had aired before data collection. The “Bully” and “Science Class” *The Real Cost* ads were launched in February 2014 [28]. The “Face of Dip” *The Real Cost* ad was launched in April 2016 in 35 targeted local markets [28]. *Tips from Former Smokers* was launched in March 2012 [20]. Finally, the *Fresh Empire* campaign was launched in October 2015 [26].

We chose these five ads since we wanted to evaluate performance of new ads (e.g., *The Real Cost*—Face of Dip, *The Real Cost*—Science Class, *Fresh Empire*—Keeping It Fresh,) and examine them in context of other ads that have already been evaluated (e.g., *The Real Cost*—Bully). We also wanted to examine the spillover effects of *Tips from Former Smokers* among adolescents.

#### 2.2.3. Attitudes toward Tobacco Products after Seeing the Ads

We assessed attitudes for the five campaign ads above by asking adolescents who recalled the ad, “After seeing or hearing this ad, did you feel more negative, more positive, or no different about tobacco products?”—as has been done in previous research [11]. Attitudes toward tobacco products for each campaign ad were assessed immediately after aided recall of the associated campaign ad. For instance, an adolescent was first asked, “In the last year, have you seen or heard any anti-smoking ads where a tiny man bullies a teenager?” If s/he reported “yes,” then h/she would then be asked, “After seeing or hearing this ad, did you feel more negative, more positive, or no different about tobacco products?”

If adolescents responded “more positive”, we asked if they could elaborate why they felt more positive. We observed that adolescents generally felt more positive about the ad itself, not tobacco products (Supplementary Table S1). Since these responses were not how we conceptualized the “more positive” response option, we dropped these observations and only analyzed the “more negative” and “no different” response options. Between 7 and 13 adolescents responded “more positive” to the five different ads (Supplementary Table S2).

#### 2.2.4. Control Variables

Survey questions included demographic characteristics: age, sex (male, female), race (White, Black or African American, other races), ethnicity (Hispanic, non-Hispanic), parental education (high school degree or less, greater than high school) and region of the country (Northeast, Midwest, South, West).

We defined cigarette smoking status at three levels: (1) not susceptible to smoking cigarettes, (2) susceptible to smoking cigarettes, and (3) current cigarette smoker—using two validated susceptibility items from Pierce et al.’s original four-item measure of adolescent smoking susceptibility—an approach used in previous research [29,30]. The two questions we used were asked of all adolescents who had not used cigarettes in the past 30 days. The questions were: “do you think you will smoke a cigarette in the next year?” and “if one of your best friends were to offer you a cigarette, would you smoke it?”. For both items, response options included: “definitely yes”, “probably yes”, “definitely not”, and “probably not”. If an adolescent chose anything but “definitely no”, then he/she was classified as susceptible to cigarette smoking. Otherwise, the adolescent was classified as not susceptible. Adolescents were defined as current smokers if they reported smoking a cigarette in the past 30 days.

Adolescents were classified as other tobacco product users if in past 30 days they had used: an e-cigarette/other vaping device; a little cigar or cigarillo; hookah; or any other tobacco product, such as chewing tobacco, dip, snus, premium cigars or any other product.

### 2.3. Data Analysis

Analyses for this study were conducted with SAS version 9.4 (SAS Institute Inc., Cary, NC, USA) to account for the complex survey design and sampling weights. We tested whether aided recall of the slogans was significantly different across the three randomized conditions (i.e., whether adolescents were randomized to report recall to *The Real Cost*, *Tips from Former Smokers*, or *Fresh Empire*) using a chi-square test. We then entered all demographic and tobacco use-related variables simultaneously in multivariable weighted logistic regression models to determine whether variables were significantly associated with: (1) aided recall of the five ads, (2) responses of negative feelings toward tobacco products after seeing or hearing each of the five ads, and (3) aided recall of each slogan. In the models where we assessed responses of negative feelings toward tobacco products, we also included increased exposure to ads (measured through the ad recall index) as a correlate. Results include weighted percentages, adjusted odds ratios (aOR) and confidence intervals (CI). We set critical α = 0.05 and used 2-tailed statistical tests. Only individuals with complete data across all relevant variables were included in analyses.

## 3. Results

### 3.1. Participant Characteristics

Adolescents (aged 13–17 years) were mostly White (72.8%) and non-Hispanic (89.9%) (see Table 1). A majority of adolescents’ parents reported greater than a high school degree (79.3%). While few adolescents were classified as current cigarette smokers (2.7%), higher numbers of adolescents were susceptible to cigarette smoking (10.4%) or reported other tobacco product use (6.7%).

### 3.2. Aided Recall of the Five Campaign Ads and Slogans

For the campaign slogans, aided recall of *The Real Cost* and *Tips from Former Smokers* slogans was high (65.5% and 71.6%, respectively), while aided recall of *Fresh Empire* was low (15.3%) (chi-square *p*-value: *p* < 0.0001).

**Figure 1:**
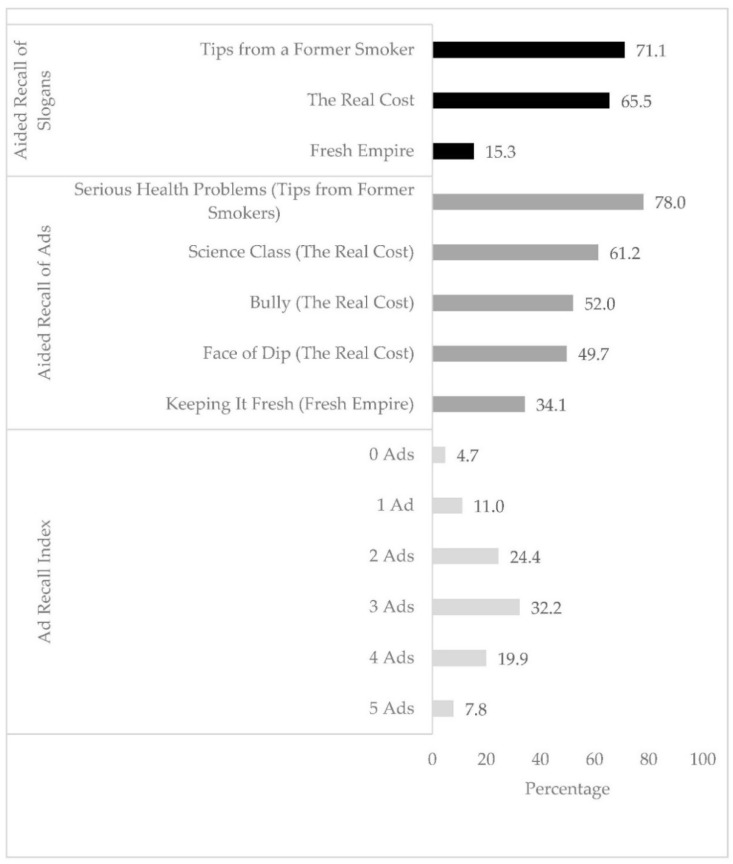
Frequencies of aided recall to slogans, ads, and ad index from a national sample of adolescents, conducted by the Survey Research Laboratory from August 2016 to May 2017.

Turning to the five campaign ads, aided recall of the *Tips from Former Smokers*—Serious Health Consequences ad was the highest (78.0%), followed by *The Real Cost*—Science Class (61.2%), *The Real Cost*—Bully (52.0%), *The Real Cost*—Face of Dip (50.0%), and *Fresh Empire*—Keeping It Fresh (34.1%) (see Figure 1). Overall, 95.3% of adolescents reported having seen or heard of at least one of the five campaign ads and 27.7% of adolescents reported having seen or heard of four or five campaign ads.

Supplementary Table S3 contains descriptive statistics on combinations of ad recall. Few adolescents (10.95%) recalled an ad from only one campaign (i.e., *The Real Cost*, *Fresh Empire*, or *Tips from Former Smokers*). The most common combination was recalling seeing one *Fresh Empire* ad and one *Tips from Former Smokers* ad (*n* = 281, 29.84%). The second and third most common combinations were recalling two *The Real Cost* ads and one *Tips from Former Smokers* ad (*n* = 215, 20.74%) and recalling two *The Real Cost* ads, one *Fresh Empire* ad, and one *Tips from Former Smokers* ad (*n* = 110, 10.37%), respectively.

### 3.3. Correlates of Aided Recall of Campaign Slogans

Some demographic and tobacco use variables were associated with aided recall of the campaign slogans (see Table 2 for full results). For brevity, only key and/or consistent results are reported in text. Female adolescents had lower odds of reporting having seen or heard of *The Real Cost* campaign slogan (aOR: 0.46, 95% CI: 0.24, 0.87) and the *Tips from Former Smokers* campaign slogan (aOR: 0.41, 95% CI: 0.21, 0.80), compared to male adolescents. Black or African American adolescents (aOR: 0.25, 95% CI: 0.10, 0.62) had lower odds of reporting having seen or heard of *The Real Cost* campaign slogan than White adolescents, but higher odds (aOR: 2.64, 95% CI 1.06, 6.54) of reporting having seen or heard of the *Fresh Empire* campaign slogan. Additionally, adolescents classified as susceptible to cigarette smoking (aOR: 3.24, 95% CI: 1.19, 8.80) had higher odds of reporting having seen or heard of the *Fresh Empire* campaign slogan, compared to adolescents not susceptible to smoking cigarettes.

### 3.4. Correlates of Aided Recall of Campaign Ads

For aided recall of the five campaign ads, few significant demographic differences among adolescents emerged (see Table 3 for full results). Of note, Black or African American adolescents (aOR: 2.28, 95% CI: 1.39, 3.73) and adolescents who had used other tobacco products in the past 30 days (aOR: 2.03, 95% CI: 1.01, 4.07) had higher odds of reporting having seen or heard of the *Fresh Empire*—Keeping It Fresh ad, compared to White adolescents and adolescents who had not used other tobacco products, respectively.

### 3.5. Attitudes toward Tobacco Products after Seeing or Hearing the Campaign Ads

Across the five ads, most adolescents reported feeling more negative toward tobacco products after having seen or heard the ads (ranging from 58% to 81.6%) (Supplementary Table S2). There were a few consistent differences in odds of reporting negative feelings toward tobacco products after having seen or heard the ads (see Table 4 for full results); however, in four of the five models, increased exposure to the ads—measured through the ad recall index—was significantly associated with higher odds of reporting negative feelings toward tobacco products. In other words, adolescents who recalled more ads had higher odds of feeling more negative toward tobacco products in response to the *The Real Cost*—Bully ad (aOR: 1.34, 95% CI: 1.01, 1.79), the *The Real Cost*—Science Class ad (aOR: 1.61; 95% CI: 1.21, 2.14), the *Fresh Empire*—Keeping It Fresh ad (aOR: 1.54, 95% CI: 1.10, 2.16), and the *Tips from Former Smokers*—Serious Health Problems ad (aOR: 1.36, 95% CI: 1.05, 1.76). For *The Real Cost*—Face of Dip ad, however, adolescents who recalled seeing more ads (beyond the *The Real Cost*—Face of Dip ad) did not have higher odds of feeling more negative toward tobacco products (aOR: 1.17, 95% CI: 0.86, 1.59).

## 4. Discussion

In this paper, we evaluated aided recall of and responses toward tobacco products from two national campaigns targeting adolescents (i.e., *The Real Cost*, *Fresh Empire*) and one campaign targeting adults (i.e., *Tips from Former Smokers*) among a critical audience—adolescents. This is noteworthy since most previous evaluations have focused on a single campaign [8,11,16,22,23,31,32], despite the fact that multiple campaigns may be concurrently reaching adolescents. In addition, we examined the impact of ads on *targeted* and *non-targeted* audiences, which is of interest because campaigns designed for one audience may have beneficial spillover effects on non-targeted audiences, increasing the impact of the campaign. Finally, this is the first paper to our knowledge that has examined aided recall of and responses toward the *Fresh Empire* campaign, as well as aided recall of and responses toward two new ads from *The Real Cost*.

Findings from our study suggest that almost all (95%) adolescents reported having seen or heard of at least one of the five tobacco campaign ads, demonstrating the ability of multiple campaigns to increase exposure to anti-tobacco messages. Interestingly, of the five ads that we examined, recall of *Tips from Former Smokers* was highest—a campaign that was not even designed to reach adolescents. Indeed, 78% of adolescents recalled the ad, whereas aided recall of the other four ads ranged from 34–61%. Additionally, more adolescents reported feeling more negative toward tobacco products after seeing or hearing the *Tips from Former Smokers* ad (82%) than any other ad.

That adult-focused educational strategies can appeal to adolescents is not new [23]; however, the magnitude of our finding is noteworthy and suggests that tobacco communication campaigns should be (1) designed with careful consideration of spillover effects and (2) evaluated to capture additional impacts beyond target audiences. Because e-cigarettes can potentially help adult smokers quit smoking, but also encourage adolescents and young adults to initiate e-cigarette use [33] which can lead to cigarette smoking [34,35,36,37,38], care designing ads about e-cigarettes, particularly with regards to spillover effects, is needed.

This is also the first study documenting that the *Fresh Empire* campaign is reaching targeted adolescents. While we were not able to assess whether recall of *Fresh Empire* was highest among its intended audience (“at-risk multicultural youth ages 12–17 who identify with hip-hop culture”) [26], we were able to show higher aided recall for African American or Black adolescents, as well as higher aided recall among youth susceptible to smoking cigarettes and among adolescents who had used other tobacco products in the past 30 days. Furthermore, we found that most youth who recalled seeing or hearing the *Fresh Empire* campaign reported feeling more negative toward tobacco products after the ad (60%). These findings therefore provide evidence of *Fresh Empire’s* reach and perceived effectiveness. It is possible that the *Fresh Empire* campaign is salient because it is tightly focused on hip hop culture. By targeting the message to a specific audience, viewers may see it as more relevant —an important consideration for attending to and thinking about a message [14,39]. Future research evaluating effectiveness and impact on outcomes are needed to fully understand the campaign mechanisms.

The two ads from *The Real Cost* for which recall was not examined in previous studies (“Science Class” and “Face of Dip”) also demonstrated high recall (>50%). Similar to the *Fresh Empire* campaign, *The Real Cost*—Face of Dip ad was targeted to a specific population (young, white males in rural regions at risk for using smokeless tobacco) [40]. While we did not find any differences in aided recall by region of the country or sex, future research could examine interactions among region, rurality, gender, and smokeless tobacco product use/susceptibility. Unfortunately, our sample was not large enough to detect potential four-way interactions, nor did we have enough specificity on type of area where participants lived (rural vs. urban). However, preliminary results from our study indicate that most adolescents report feeling more negative toward tobacco products after seeing these ads (71–79%). An evaluation of this campaign’s impact on its target audience is currently underway by the FDA.

Overall, these findings suggest that the ads and slogans from FDA’s *The Real Cost* and CDC’s *Tips from Former Smokers*, which emphasize the negative health effects of smoking, harmful chemicals in cigarettes and other tobacco products, and addiction, are both reaching adolescents with separate but likely additive message themes [41]. Indeed, among adolescents who recalled the ads, the highest percentage of negative feelings toward tobacco products was reported for *Tips from Former Smokers* and “Face of Dip”—both of which convey the harmful consequences of tobacco use. Moreover, we found that increasing exposure to ads (or in other words, higher dose) was associated with feeling more negative toward tobacco products in response to many of the ads. Thus, greater cumulative cross-campaign exposure to ads with different themes and messaging strategies may be associated with better outcomes, an important point that may be missed by evaluations of single campaigns.

### Limitations

This study had a number of limitations. First, we did not examine recall of all ads featured in the campaigns we studied, nor did we examine other campaigns, such as *Truth*. It is possible that participants were exposed to and remembered campaign ads that we did not ask about in our study. Second, we only used one item to evaluate perceptions of the ads (attitudes toward tobacco products). Although this measure has been used in previous research [11], findings indicated that adolescents misunderstood the “more positive” response option. We therefore dropped the “more positive” responses; however, sensitivity analysis reveals that including these participants did not change results (see Supplementary Table S4). Additional measures, such as perceived message effectiveness, should be examined. Third, we examined aided—not unaided—recall of ads. Fourth, we did not assess frequency of exposure to individual ads. Differences in recall are likely associated with the varying resources and approaches—such as media buys (per market), dissemination channels, and launch dates—of these campaigns.

Fifth, given our data collection method (i.e., phone), adolescents were not able to actually see or hear the ads, or see the slogans. Presumably, showing participants the ads and slogans would have increased recall even further. Relatedly, it is possible that parents or guardians were present in the room when adolescents were providing results. To assess issues related to phone privacy, we examined two questions at the end of the survey that asked about whether an adult was in the room during survey completion. 362 adolescents (37.8% of the sample) reported that an adult was in the room “at any point while you were answering these questions.” However, of these individuals, only 8 (2.7%) indicated that their answers would have been different without the adult in the room. Nevertheless, social desirability (resulting from either presence of adults or because of the interviewers) could have biased results.

Sixth, it is possible that our description of the *Tips from Former Smokers*—Serious Health Consequences ad was more general than descriptions of the other ads. Therefore, even though adolescents reported having seen or heard the ad, it may have been one of a few different ads from *Tips from Former Smokers* (or perhaps another campaign). However, in aided recall of slogans where each campaign was explicitly named, aided recall of *Tips from Former Smokers* was higher than aided recall of *The Real Cost* and *Fresh Empire*, suggesting that adolescents had high exposure to the campaign. Finally, we randomized adolescents to hear 1 of 3 slogans, instead of instead of answering questions about all 3 slogans. This reduced the sample size and power for recall of each slogan.

## 5. Conclusions

We found that tobacco communication campaigns, specifically *The Real Cost*, *Tips from Former Smokers*, and *Fresh Empire* are reaching US adolescents using different audience targeting strategies. These data support CDC and FDA messaging strategies for adolescents, including campaigns targeted toward them as well as non-targeted campaigns to which they are exposed. Future work should examine how campaigns can work individually and cumulatively to impact adolescents’ tobacco-related attitudes, beliefs, and behaviors, ultimately leading to healthier adolescents and a healthier nation.

## Figures and Tables

**Table 1:** Participant characteristics from a national sample of adolescents, conducted by the Carolina Survey Research Laboratory from August 2016 to May 2017, *n* = 975.

Variable	Unweighted *n*	Weighted % or Mean (Standard Error)
Sex at birth		
Male	481	50.10
Female	491	49.90
Age, mean (SE)	975	15.06 (0.06)
Race		
White	788	72.81
Black or African American	123	16.88
Other	64	10.31
Ethnicity		
Non-Hispanic	913	89.88
Hispanic	61	10.12
Parent Education		
Greater than high school	770	79.30
High school or less	200	20.70
Cigarette smoking status		
Not susceptible	845	86.91
Susceptible	102	10.36
Current cigarette smoker	27	2.74
Other tobacco product use		
No	901	93.31
Yes	74	6.69
Region		
Northeast	108	17.38
Midwest	219	21.91
South	549	37.14
West	98	23.57

**Table 2:** Weighted logistic regression results for aided recall of slogans from a national sample of adolescents, conducted by the Carolina Survey Research Laboratory from August 2016 to May 2017 ^1^.

	*The Real Cost*, *n* = 316		*Tips from Former Smokers*, *n* = 322		*Fresh Empire*, *n* = 337	
Variable	*n* who recalled seeing the slogan/total *n* (weighted %)	aOR (95% CI)	*n* who recalled seeing the slogan/total *n* (weighted %)	aOR (95% CI)	*n* who recalled seeing the slogan/total *n* (weighted %)	aOR (95% CI)
Sex at birth						
Male	111/154 (73.71)	REF	116/153 (80.89)	REF	20/174 (14.90)	REF
Female	96/161 (56.69)	**0.46 (0.24, 0.87)**	108/168 (62.12)	**0.41 (0.21, 0.80)**	24/162 (15.93)	1.01 (0.47, 2.21)
Age	--	0.96 (0.75, 1.24)	--	1.05 (0.84, 1.31)	--	0.89 (0.67, 1.17)
Race						
White	181/258 (72.55)	REF	179/256 (71.62)	REF	32/274 (12.00)	REF
Black or African American	16/39 (36.48)	**0.25 (0.10, 0.62)**	33/44 (75.71)	1.28 (0.50, 3.28)	10/40 (29.85)	**2.64 (1.06, 6.54)**
Other	11/19 (56.98)	0.49 (0.14, 1.69)	13/22 (58.61)	0.38 (0.13, 1.15)	2/23 (14.54)	0.57 (0.08, 4.16)
Ethnicity						
Non-Hispanic	196/ 299 (65.37)	REF	210/297 (70.66)	REF	41/317 (13.96)	REF
Hispanic	11/16 (66.54)	1.29 (0.25, 6.49)	15/25 (74.74)	2.08 (0.64, 6.76)	3/20 (27.67)	1.80 (0.40, 8.13)
Parent Education						
Greater than high school	166/244 (67.04)	REF	182/261 (71.67)	REF	33/265 (15.22)	REF
High school or less	41/71 (59.54)	0.66 (0.29, 1.50)	42/60 (68.43)	0.96 (0.39, 2.36)	11/69 (16.55)	0.95 (0.34, 2.63)
Cigarette Smoking Status						
Not susceptible	179/274 (64.80)	REF	196/277 (73.46)	REF	36/294 (13.34)	REF
Susceptible	20/30 (72.47)	0.97 (0.29, 3.27)	24/36 (57.83)	0.67 (0.26, 1.71)	7/36 (31.02)	**3.24 (1.19, 8.80)**
Current cigarette smoker	8/11 (63.77)	0.61 (0.10, 3.83)	5/9 (46.62)	0.48 (0.07, 3.31)	1/7 (9.84)	1.02 (0.04, 25.99)
Other tobacco product use						
No	185/287 (64.75)	REF	212/302 (71.63)	REF	41/312 (15.10)	REF
Yes	23/29 (74.68)	1.56 (0.40, 6.06)	13/20 (60.53)	0.56 (0.14, 2.34)	3/25 (17.68)	2.40 (0.29, 20.03)
Region						
Northeast	16/24 (61.40)	REF	40/51 (82.67)	REF	10/33 (36.68)	REF
Midwest	43/63 (69.70)	1.43 (0.38, 5.37)	44/72 (62.75)	**0.36 (0.13, 0.98)**	4/84 (4.27)	**0.09 (0.02, 0.31)**
South	122/186 (61.60)	1.24 (0.36, 4.29)	124/168 (74.94)	0.62 (0.24, 1.61)	29/195 (17.89)	**0.39 (0.16, 0.99)**
West	26/42 (67.17)	1.26 (0.34, 4.64)	17/31 (60.76)	0.33 (0.10, 1.08)	1/25 (4.82)	**0.09 (0.01, 0.75)**

^1^ Boldface indicates significance *p* < 0.05.

**Table 3:** Weighted logistic regression results for aided recall of the five ads from a national sample of adolescents, conducted by the Carolina Survey Research Laboratory from August 2016 to May 2017 ^1^.

	*The Real Cost*—Bully, *n* = 965	*The Real Cost*—Science Class, *n* = 966	*The Real Cost*—Face of Dip, *n* = 965	*Tips from Former Smokers*—Serious Health Problems, *n* = 965	*Fresh Empire*—Keeping It Fresh, *n* = 966
Variable	aOR (95% CI)	aOR (95% CI)	aOR (95% CI)	aOR (95% CI)	aOR (95% CI)
Sex at birth					
Male	REF	REF	REF	REF	REF
Female	0.93 (0.66, 1.30)	0.72 (0.51, 1.02)	0.76 (0.54, 1.08)	0.92 (0.58, 1.47)	0.96 (0.67, 1.39)
Age	1.01 (0.89, 1.15)	0.95 (0.84, 1.09)	1.01 (0.89, 1.15)	**1.25 (1.06, 1.49)**	1.10 (0.96, 1.26)
Race					
White	REF	REF	REF	REF	REF
Black or African American	1.09 (0.68, 1.76)	0.85 (0.52, 1.39)	0.81 (0.50, 1.32)	0.88 (0.47, 1.62)	**2.28 (1.39, 3.73)**
Other	0.65 (0.32, 1.34)	0.62 (0.30, 1.27)	0.88 (0.43, 1.82)	0.52 (0.22, 1.25)	1.67 (0.83, 3.38)
Ethnicity					
Non-Hispanic	REF	REF	REF	REF	REF
Hispanic	1.02 (0.48, 2.14)	1.91 (0.86, 4.23)	1.10 (0.52, 2.31)	1.15 (0.48, 2.75)	1.69 (0.80, 3.59)
Parent Education					
Greater than high school	REF	REF	REF	REF	REF
High school or less	0.90 (0.59, 1.38)	0.89 (0.59, 1.36)	1.07 (0.69, 1.66)	**0.52 (0.31, 0.87)**	1.16 (0.73, 1.85)
Cigarette Smoking Status					
Not susceptible	REF	REF	REF	REF	REF
Susceptible	0.89 (0.50, 1.58)	1.24 (0.69, 2.20)	1.30 (0.73, 2.31)	1.66 (0.69, 3.98)	0.95 (0.52, 1.74)
Current cigarette smoker	0.61 (0.20, 1.82)	3.46 (0.32, 1.54)	0.75 (0.26, 2.19)	N/A	0.38 (0.11, 1.33)
Other tobacco product use					
No	REF	REF	REF	REF	REF
Yes	1.66 (0.80, 3.47)	0.70 (0.32, 1.54)	0.70 (0.34, 1.41)	1.01 (0.40, 2.51)	**2.03 (1.01, 4.07)**
Region					
Northeast	REF	REF	REF	REF	REF
Midwest	1.18 (0.68, 2.05)	1.25 (0.71, 2.12)	1.00 (0.58, 1.74)	0.93 (0.45, 1.92)	0.74 (0.40, 1.35)
South	1.06 (0.65, 1.72)	1.35 (0.83, 2.27)	1.24 (0.76, 2.03)	1.48 (0.78, 2.82)	1.04 (0.62, 1.77)
West	1.18 (0.60, 2.09)	1.27 (0.69, 2.45)	0.58 (0.31, 1.08)	0.70 (0.33, 1.51)	1.30 (0.68, 2.48)

^1^ Boldface indicates significance *p* < 0.05.

**Table 4:** Weighted logistic regression results for negative feelings toward tobacco products among adolescents who reported having seen or heard each ad, from a national sample of adolescents, conducted by the Carolina Survey Research Laboratory from August 2016 to May 2017 ^1^.

	*The Real Cost*—Bully, *n* = 480	*The Real Cost*—Science Class, *n* = 586	*The Real Cost*—Face of Dip, *n* = 511	*Tips from Former Smokers*—Serious Health Problems, *n* = 786	*Fresh Empire*—Keeping It Fresh, *n* = 307
Variable	aOR (95% CI)	aOR (95% CI)	aOR (95% CI)	aOR (95% CI)	aOR (95% CI)
Ad recall index	**1.34 (1.01, 1.79)**	**1.61 (1.21, 2.14)**	1.17 (0.86, 1.59)	**1.36 (1.05, 1.76)**	**1.54 (1.10, 2.16)**
Sex at birth					
Male	REF	REF	REF	REF	REF
Female	0.73(0.42, 1.24)	1.18 (0.70, 1.99)	0.67 (0.35, 1.28)	1.24 (0.74, 2.07)	**2.01 (1.05, 3.84)**
Age	1.07 (0.89, 1.29)	0.98 (0.81, 1.19)	1.08 (0.87, 1.36)	**1.31 (1.08, 1.57)**	1.03 (0.79, 1.35)
Race					
White	REF	REF	REF	REF	REF
Black or African American	0.73 (0.35, 1.53)	1.13 (0.56, 2.30)	0.49 (0.22, 1.10)	0.77 (0.39, 1.55)	1.74 (0.70, 4.36)
Other	2.13 (0.47, 9.58)	0.52 (0.18, 1.50)	0.89 (0.25, 3.14)	1.16 (0.34, 3.93)	1.21 (0.39, 3.81)
Ethnicity					
Non-Hispanic	REF	REF	REF	REF	REF
Hispanic	1.35 (0.37, 4.97)	0.97 (0.36, 2.64)	**10.60 (1.32, 85.36)**	0.60 (0.22, 1.66)	1.39 (0.39, 5.00)
Parent Education					
Greater than high school	REF	REF	REF	REF	REF
High school or less	1.43 (0.75, 2.75)	1.06 (0.58, 1.96)	0.72 (0.36, 1.42)	1.13 (0.60, 2.12)	1.20 (0.54, 2.67)
Cigarette Smoking Status					
Not susceptible	REF	REF	REF	REF	REF
Susceptible	1.43 (0.61, 3.35)	0.97 (0.42, 2.22)	0.71 (0.27, 1.42)	0.56 (0.26, 1.20)	0.34 (0.11, 1.06)
Current cigarette smoker	2.07 (0.29, 14.62)	1.36 (0.33, 5.62)	4.08 (0.36, 46.62)	0.29 (0.08, 1.01)	0.80 (0.08, 8.55)
Other tobacco product use					
No	REF	REF	REF	REF	REF
Yes	**0.36 (0.15, 0.90)**	**0.36 (0.13, 0.95)**	0.58 (0.20, 1.68)	0.93 (0.38, 2.29)	2.51 (0.76, 8.28)
Region					
Northeast	REF	REF	REF	REF	REF
Midwest	0.79 (0.34, 1.86)	0.69 (0.31, 1.55)	0.44 (0.15, 1.27)	0.70 (0.30, 1.66)	0.71 (0.26, 1.99)
South	1.33 (0.60, 2.98)	0.85 (0.40, 1.79)	0.54 (0.19, 1.51)	0.83 (0.37, 1.87)	0.90 (0.35, 2.26)
West	1.07 (0.41, 2.80)	0.89 (0.34, 2.32)	0.48 (0.12, 2.00)	0.68 (0.25, 1.83)	2.54 (0.79, 8.20)

^1^ Boldface indicates significance *p* < 0.05.

## Supplementary Materials

The following are available online at http://www.mdpi.com/1660-4601/15/11/2363/s1, Table S1: Free text responses to the ads among individuals who reported that they feel more positive toward tobacco products after reporting having seen or heard the ad, Table S2: Descriptive statistics for attitudes toward tobacco products after seeing the ad among adolescents who recalled seeing the ad, Table S3: Combinations of aided recall to ads, *n* = 973, Table S4: Weighted logistic regression results for negative feelings toward tobacco products among adolescents who reported having seen or heard each ad, from a national sample of adolescents, conducted by the Carolina Survey Research Laboratory from August 2016 to May 2017.
