# Supplementary material for: Adolescents’ Aided Recall of Targeted and Non-Targeted Tobacco Communication Campaigns in the United States
Source: Int J Environ Res Public Health. 2018 Oct 25;15(11):2363. doi: 10.3390/ijerph15112363 (PMC6266583; doi:10.3390/ijerph15112363)
Supplement: Supplementary file 1 [file ijerph-15-02363-s001.pdf]

Supplementary Materials

Table S1. Free text responses to the ads among individuals who reported that they feel more positive toward tobacco products after reporting having seen or heard the ad (Exact question wording: "Can you tell me in a few words why you felt more positive?").

The Real Cost—Bully	Bad for you
	Encouraged not to smoke
	Gave more info about tobacco
	Grandfather smokes & gives hope that he might stop
	I really don't want to smoke
	It looked convincing
	It seemed like that tobacco was it's very bad for teenagers it seemed like, it seemed like they're willing to sell to anybody that will take their money
	Leads to cancer
	Like to see people trying to stop smoking
	More positive because they were putting out more anti-smoking ads to educate the public about the harms of smoking
	Tobacco is bad for you and your lungs
The Real Cost—Science Class	Bad for you
	Because she would never smoke
	Don't know
	It did creep her out
	Leave it alone
	More against it
	Showed what it can do to you
	Shows you to not smoke
	Tobacco is like a bug and it controls you
	Trying to reach out to kids that do smoke
	Visual of what it could do to you
The Real Cost—Face of Dip	Ad showed what it could do to your mouth
	Because cigarettes make you sick
	Because it was scary
	He doesn't know
	Medical reasons, harmful
	Mess your teeth up
	Nobody in family dips
Tips from Former Smokers—Serious Health Problems	After seeing that ad, it made me think more of why you should not smoke
	Because it shows the terrible things that could happen if you smoke, this discourages ppl from wanting to smoke
	Cigarettes hurt your body
	I don't know
	Make your lungs bad
	Makes you not want to do those negative things
	Not really
	Safety and harmful
	Stop more people from smoking
	Testimony to the truth about smoking
Fresh Empire—Keeping It Fresh	Because she showed how much she cared about her family and how much she wanted to protect them from tobacco.
	Because they rejected it
	Because they were showing that it is possible to give up smoking
	He did the right thing to not harm him or his family
	I agree that smoking causes problems, grandma died from it.
	I just felt more positive; its hard to describe
	It was showing people not to smoke
	Not to smoke because it's bad for you
	Positive that they were saying no
	Positive is good, I don't know
	Smoking will kill you

Table S2. Descriptive statistics for attitudes toward tobacco products after seeing the ad among adolescents who recalled seeing the ad.

Variable	Unweighted n	Weighted %
The Real Cost—Bully		
More negative	291	58.00
More positive	11	2.99
No different	195	39.00
The Real Cost—Science Class		
More negative	433	71.74
More positive	11	1.61
No different	160	26.65
The Real Cost—Face of Dip		
More negative	419	79.49
More positive	7	1.63
No different	94	18.87
Tips from Former Smokers—Serious Health Problems		
More negative	655	81.57
More positive	13	1.62
No different	134	16.82
Fresh Empire—Keeping It Fresh		
More negative	194	60.37
More positive	12	3.49
No different	114	36.15
Note: Cases where adolescents answered "do not know" or "refuse to answer" were coded as missing and dropped from analyses. In logistic regression models, responses of "more positive" were dropped from analyses.		

Table S3. Combinations of aided recall to ads, n=973.

Ad Recall Index	Unweighted n	Weighted %
Recalled 0 ads, n=40	40	4.71
Recalled 1 ad, n=104		
1 *The Real Cost* ad	34	5.00
1 *Fresh Empire* ad	8	0.65
1 *Tips from Former Smokers* ad	62	5.30
Recalled 2 ads, n=218		
2 *The Real Cost* ads	35	4.87
1 *The Real Cost* ads, 1 *Fresh Empire* ad	11	1.48
1 *The Real Cost* ad, 1 *Tips from Former Smokers* ad	142	12.25
1 *Fresh Empire* ad, 1 *Tips from Former Smokers* ad	281	29.84
Recalled 3 ads, n=317		
3 *The Real Cost* ads	23	3.08
2 *The Real Cost* ads, 1 *Fresh Empire* ad	15	1.59
2 *The Real Cost* ads, 1 *Tips from Former Smokers* ad	215	20.74
1 *The Real Cost* ad, 1 *Fresh Empire* ad, 1 *Tips from Former Smokers* ad	64	6.83
Recalled 4 ads, n=217		
3 *The Real Cost* ads, 1 *Fresh Empire* ad	6	0.59
3 *The Real Cost* ads, 1 *Tips from Former Smokers* ad	101	8.88
2 *The Real Cost* ads, 1 *Fresh Empire* ad, 1 *Tips from Former Smokers* ad	110	10.37
Recalled 5 ads, n=77	77	7.82
Note: All categories are mutually exclusive and percentages add to 100%.		

Table S4. Weighted logistic regression results for negative feelings toward tobacco products among adolescents who reported having seen or heard each ad, from a national sample of adolescents, conducted by the Carolina Survey Research Laboratory from August 2016 to May 2017 [a,b]

	The Real Cost—Bully, n=490	*The Real Cost*—Science Class, n=595	*The Real Cost*—Face of Dip, n=517	*Tips from Former Smokers*—Serious Health Problems, n=797	*Fresh Empire*—Keeping It Fresh, n=319
**Variable**	**aOR (95% CI)**	**aOR (95% CI)**	**aOR (95% CI)**	**AOR (95% CI)**	**AOR (95% CI)**
Ad recall index	**1.35 (1.01, 1.81)**	**1.58 (1.19, 2.08)**	1.23 (0.91, 1.66)	**1.39 (1.09, 1.79)**	**1.52 (1.09, 2.10)**
Sex at birth					
Male	REF	REF	REF	REF	REF
Female	0.75 (0.44, 1.26)	1.18 (0.71, 1.96)	0.73 (0.39, 1.35)	1.34 (0.82, 2.21)	1.58 (0.85, 2.92)
Age	1.09 (0.90, 1.31)	0.98 (0.81, 1.19)	1.11 (0.90, 1.38)	**1.32 (1.10, 1.58)**	1.02 (0.79, 1.32)
Race					
White	REF	REF	REF	REF	REF
Black or African American	0.64 (0.31, 1.32)	1.15 (0.58, 2.29)	**0.46 (0.22, 0.99)**	0.73 (0.37, 1.41)	1.22 (0.53, 2.81)
Other	2.22 (0.49, 10.02)	0.52 (0.19, 1.43)	0.82 (0.26, 2.59)	0.85 (0.30, 2.41)	1.30 (0.41, 4.11)
Ethnicity					
Non-Hispanic	REF	REF	REF	REF	REF
Hispanic	1.25 (0.35, 4.44)	0.95 (0.36, 2.49)	**5.64 (1.24, 25.63)**	0.64 (0.24, 1.72)	1.35 (0.40, 4.61)
Parent Education					
Greater than high school	REF	REF	REF	REF	REF
High school or less	1.45 (0.77, 2.73)	1.02 (0.57, 1.82)	0.68 (0.35, 1.30)	1.03 (0.56, 1.88)	1.08 (0.52, 2.25)
Cigarette Smoking Status					
Not susceptible	REF	REF	REF	REF	REF
Susceptible	1.46 (0.63, 3.40)	1.01 (0.44, 2.30)	0.66 (0.27, 1.62)	0.60 (0.28, 1.26)	0.36 (0.12, 1.07)
Current cigarette smoker	2.22 (0.32, 15.24)	1.40 (0.35, 5.71)	4.62 (0.40, 53.71)	0.33 (0.09, 1.24)	0.89 (0.09, 8.65)
Other tobacco product use					
No	REF	REF	REF	REF	REF
Yes	**0.37 (0.15, 0.92)**	**0.38 (0.14, 0.98)**	0.58 (0.20, 1.64)	0.97 (0.39, 2.40)	2.45 (0.75, 7.97)
Region					
Northeast	REF	REF	REF	REF	REF
Midwest	0.73 (0.31, 1.74)	0.65 (0.29, 1.46)	0.40 (0.14, 1.17)	0.67 (0.28, 1.58)	0.87 (0.32, 2.36)
South	1.16 (0.51, 2.62)	0.76 (0.36, 1.60)	0.47 (0.17, 1.32)	0.69 (0.31, 1.54)	0.94 (0.39, 2.26)
West	1.04 (0.39, 2.73)	0.89 (0.34, 2.30)	0.48 (0.12, 2.01)	0.66 (0.25, 1.78)	2.52 (0.81, 7.85)

[a] Boldface indicates significance $p < 0.05$; [b] "More negative" responses are compared to "more positive" and "no difference" responses. Red indicates that the results presented here either became significant or non-significant, compared to results in Table 3.
